# Fluoxetine suppresses inflammatory reaction in microglia under OGD/R challenge via modulation of NF-κB signaling

**DOI:** 10.1042/BSR20181584

**Published:** 2019-04-26

**Authors:** Mouli Tian, Mei Yang, Zhenjie Li, Yiru Wang, Wei Chen, Liye Yang, Yonghua Li, Hongbin Yuan

**Affiliations:** Department of Anesthesiology, Changzheng Hospital, Second Military Medical University, Shanghai, China

**Keywords:** fluoxetine, inflammation, microglia, NF-κB, OGD/R

## Abstract

We aimed to investigate the anti-inflammatory role of fluoxetine, a selective serotonin reuptake inhibitor, in microglia (MG) and the mechanisms under oxygen glucose deprivation/reoxygenation (OGD/R). An OGD/R model on BV-2 cells was used for the study of microglia under ischemia/reperfusion injury in ischemic stroke. Lentiviral transfection was applied to knock down IκB-α. Enzyme-linked immunosorbent assay (ELISA) was used for detecting levels of TNF-α, IL-1β, and IL-6, and real-time PCR was used to assess the expression of IκB-α protein. Western blotting was applied to analyze NF-κB-signaling related proteins and Cell Counting Kit-8 (CCK-8) was used for assessing cell viability. Molecular docking and drug affinity responsive target stability (DARTS) assay were used for the detection of the interaction between IκB-α and fluoxetine. We found that fluoxetine decreased the levels of TNF-α, IL-1β, and IL-6 in supernatant as well as NF-κB subunits p65 and p50 in BV-2 cells under OGD/R. Fluoxetine significantly increased the level of IκB-α through the inhibition of IκB-α ubiquitylation and promoted the bonding of IκB-α and fluoxetine in BV-2 cells under OGD/R. Knocking down IκB-α attenuated the decreasing effect of TNF-α, IL-1β, and IL-6 as well as p65 and p50 in BV-2 cells under OGD/R led to by fluoxetine. In conclusion, our present study demonstrated the anti-inflammatory role of fluoxetine and its mechanisms related to the modulation of NF-κB-related signaling in MG under ischemia/reperfusion challenge.

## Introduction

Ischemic stroke is one of the common age-related diseases worldwide [1–3]. On the occurrence of ischemic stroke, rapture or blockage of blood vessels causes obstructed circulation, resulting in hypoxia injury of cerebral cells and tissues [4]. The main therapeutic approach is to recover blood and nutrient supply, which, if applied in time, will effectively alleviate the severity of ischemic stroke. However, this traditional therapy has been increasingly questioned by modern clinical studies, which pointed out several related adverse effects [5,6]. Circulation recovery, commonly referred to as reperfusion, has been considered to lead to cerebral inflammatory responses via the activation of microglia (MG) after ischemia. Activated MG secretes several kinds of proinflammatory cytokines, leading to the detrimental effect on neurons in cell viability and promoting neuronal apoptosis [7,8]. Consequently, suppressing the inflammatory responses triggered by the activation of MG under ischemia/reperfusion challenge might provide a potential therapeutic pathway in the alleviation of damage of neuronal function under cerebral ischemia [9–11].

MG extensively distribute in central nervous system, accounting for approximately one out of five of total gliocytes in brain [12]. MG are regarded as macrophages in central nervous system judged from characteristics and biological activity [13–15]. For example, the activation of MG secretes a variety of inflammatory cytokines like macrophages, thus playing a dual role in neural injury and recovery [16]. On the occurrence of cerebral ischemia, MG are mobilized and transferred to the damaged areas for the maintenance of basic neural functions [17]. However, cerebral reperfusion may further activate MG and lead to the enhancement of a cascade inflammatory response, ultimately resulting in further injury and malfunction of neurons [18,19]. So far, since seldom ideal drugs are available, it is urgent for the development of novel therapeutic strategies against ischemic stroke.

Fluoxetine, a selective serotonin reuptake inhibitor, is mainly used in the treatment of major depressive disorder, obsessive-compulsive disorder, panic disorder, bulimia, binge eating disorder, premenstrual dysphoric disorder, and bipolar depression [20]. However, it has been uncovered by recent studies demonstrating that fluoxetine is beneficial in improving neurological functions after cerebral ischemic injury due to its function in protecting neurons against ischemic injury and promoting neuron regeneration [21–23]. The positive effect of fluoxetine in the recovery of patients with cerebral stroke has been proven in clinical studies. Several studies noted that fluoxetine protected neurons via reducing the inflammatory responses triggered by MG [24,25]. However, to ultimately take advantage of the anti-inflammatory effect of fluoxetine against ischemia-reperfusion injury, the specific mechanisms should be clarified.

In the present study, we used oxygen glucose deprivation/reoxygenation (OGD/R) models for the study of ischemia-reperfusion injury and BV-2 cell line for MG. We investigated the anti-inflammatory effect of fluoxetine in MG under OGD/R challenge and its signaling pathway related to NF-κB. We believe that the present study might provide strong evidence for applying fluoxetine in the development as a novel drug against ischemic stroke.

## Materials and methods

### Cell culture and treatment

BV-2 cells were purchased from the Cell Bank of the Chinese Academy of Sciences. During experiments, cells were cultured in Dulbecco’s modification of Eagle’s medium (DMEM; Gibco, Grand Island, NY, U.S.A.) supplemented with 10% fetal calf serum (FCS; Gibco) at 37°C under 5% CO_2_ and 0.25% typsin was used for cell generation at the coverage of 70%. Cells subjected to OGD/R challenge were kept in glucose-free DMEM (Gibco) under 5% CO_2_ and 95% N_2_ for 2 h followed by 48-h culture under normal condition. 293TN cells, obtained from ATCC, were cultured in DMEM supplemented with 10% FCS at 37°C under 5% CO_2_.

### Source of drug and cell viability test

Fluoxertine hydrochloride (34.6 mg; Sigma–Aldrich, St. Louis, MO, U.S.A.) was dissolved in 10 ml DMSO to get 10 mM stock solution, followed by filtered through a 0.22 μm membrane filter, and stored at 4°C. BV-2 cells were seeded into 96-well plates in the density of 1 × 10^5^ cells/well. During experiments, fluoxetine at different concentrations (1, 2, 5, 10, and 20 μM) were administrated to BV-2 cells under the challenge of OGD/R at 2 h before OGD/R treatment. Cell Counting Kit-8 (CCK-8) assay (Dojindo, Kamimashiki-gun Kumamoto, Japan) was conducted for the analysis of cell viability through the addition of 10 μl CCK-8 solution in each well. After incubated at 37°C under 5% CO_2_ for 4 h, cells were measured in absorbance by Multiskan GO (Thermo Scientific, Pittsburgh, PA, U.S.A.) at 450 nm. The maximum concentration of fluoxertine without significant cytotoxicity was calculated and used for subsequent experiments.

### Molecular docking

Molecular docking was conducted as previously described [26,27]. The IκB-α structure was obtained from the PDB database. All water molecules as well as ions and endogenous ligands were removed and hydrogen was added through the LePro program of LeDock. The pH value was adjusted to 7 by adding hydrogen to the polar amino residues. The 2D structure of fluoxetine was translated into a 3D structure for molecular docking using the Corina program. LeDock was used for the detection of molecular docking. The binding sites included all atoms of proteins within 4Å of the endogenous ligands. The potential conformations were searched based on the simulated annealing and genetic algorithm. The fluoxetine binding site, binding direction, and groups with corresponding molecular conformations were searched in the selected region. Pymold was used to visualize the conformation of molecular docking.

### DARTS experiment

DARTS experiments were carried out as described previously [28]. In brief, recombinant IκB-α (10 μg) was dissolved in 1 ml sterile water together with 10 μl Halt Protease and Phosphatase Inhibitor Cocktail. The mixture was put on ice and pipetted into two microtubes with 300 μl for each one and incubated at room temperature for 10 min. One of the two tubes was added with 3 μl 100 mM fluoxetine, while the other one with 3 μl sterile water. The contents were mixed thoroughly by tapping the tube, and the mixture was centrifuged and incubated at 25°C for 1 h. The solution was then pipetted into three tubes with 45 μl for each one, together with 1 × TNC and pronase (10 mg/ml), incubated at 25°C for 30 min. Protein-loading buffer was then added and the final mixture was boiled for 5 min. The level of residual IκB-α was detected by western blotting.

### Lentivirus packaging and transfection

The siRNA sequences (5′-GTCATTGGTCAGGTGAAGG-3′) complementarily binding to IκB-α (NM_010907.2) was chosen, which were homologous to IκB-α. The oligonucleotide templates of those shRNAs were chemically synthesized and cloned into the linear pSIH1-H1-copGFP shRNA Vector (System Biosciences, Palo Alto, CA, U.S.A.). The oligonucleotide templates were obtained through digestion by BamHI and EcoRI (Takara, Otsu, Shiga, Japan) and purified by agarose gel electrophoresis. The invalid siRNA sequences (5′-GAAGCCAGATCCAGCTTCC-3′) were used as the negative control (NC). Sequencing was used to confirm the constructed vectors (pSIH1-shRNA-IκB-α and pSIH1-NC). Cells in the absence of lentivirus transfection was used as the control group and those transfected with lentivirus vector was applied as the Lv-Control group. For lentivirue transfection, 293TN cells were seeded in 10-cm dishes. Each pSIH1-shRNA-IκB-α vector or pSIH1-NC (2 μg) and pPACK Packaging Plasmid Mix (10 μg; System Biosciences) were added into the cultural medium (DMEM supplemented with 10% FCS) along with Lipofectamine 2000 (Invitrogen, Carlsbad, CA, U.S.A.) in accordance with the manufacturer’s protocol. After cultivation for 8 h, the supernatant was harvested and then cleared by centrifugation at 5000 × ***g*** at 4°C for 5 min and passed through a 0.45 µm PVDF membrane (Millipore, Billerica, MA, U.S.A.). The titer of virus was determined by gradient dilution. The packaged lentivirus was named as Lv-shRNA-IκB-α and Lv-NC accordingly in the description of the results. The transfection efficiency of lentivirus was determined through the comparison of microscopy images obtained from the dark field and fluorescent scope.

### Enzyme-linked immunosorbent assay

BV-2 cells were seeded into a 96-well plate in the density of 5 × 10^4^ cells/well. Cells were challenged under OGD/R and supernatant was extracted and quantitated using the bicinchoninic acid method (BCA, Thermo Scientific) method. Intracellular TNF-α, IL-1β, and IL-10 were measured using mouse TNF-α, IL-1β, and IL-10 ELISA kits (Invitrogen) according to the manufacturer’s instructions. The absorbent values were obtained on Multiskan GO at 450 nm.

### Realtime PCR

BV-2 cells were seeded into a six-well plate in the density of 3 × 10^5^ cells/well and challenged with OGD/R. Total RNA from BV-2 cells was isolated by TRIzol (Invitrogen). The first-strand cDNA was synthesized using PrimeScript RT Master Mix (Takara). The 7500 Real Time PCR System and the Fast Start Universal SYBR Green Master (Roche, Basel, Switzerland) were used for real-time PCR. The PCR primers were listed as following: β-actin forward, 5′- AACCCTAAGGCCAACCGTGAAAAG-3′, β-actin reverse 5′-TGGCGTGAGGGAGAGCATAGC-3′; IκB-α forward, 5′-CCGTCCTGCAGGCCACCAACTACA-3′, IκB-α reverse, 5′-CAAGAGCGAAACCAGGTCAGGATT-3′.

### Western blotting

Total protein was extracted from BV-2 cells using M-PER mammalian protein extraction reagent (Pierce, IL, U.S.A.). The equal amount of protein (20 μg per lane) estimated by a BCA protein assay kit (Pierce) was loaded onto 11% SDS-PAGE gels and transferred onto nitrocellulose membranes. The blots were probed with a monoclonal antibody against mouse anti-P65 (1:500), anti-P-p65 (1:500), anti-P50 (1:500), anti-P-p50 (1:500), anti-IkB-α (1:500), and anti-β-actin (1:2000) (Santa Cruz Biotechnology, Dallas, TX, U.S.A.), followed by the incubation of secondary HRP-conjugated anti-mouse/rabbit antibody (1:2000; Santa Cruz). After washed, the bands were detected by chemiluminescence and imaged with X-ray films. β-actin was used as an endogenous reference for normalization.

### Data analysis

All data were expressed as means ± S.D. Statistical analysis was conducted using Student’s *t* test or one-way analysis of variance followed by least significant difference test for the comparison of two independent groups or multiple comparisons, respectively. A *P*-value <0.05 was considered statistically significant. All statistical analysis was performed using SPSS 13.0 software (SPSS, Chicago, IL, U.S.A.).

## Results

### Administration of fluoxetine suppresses inflammatory reaction in an OGD/R model of BV-2 cells

**Figure 1:**
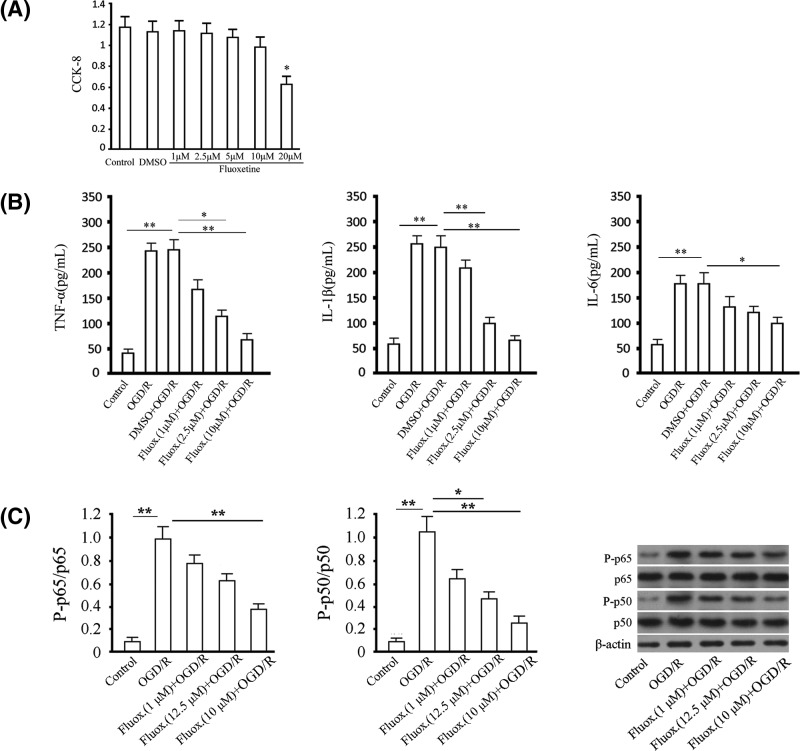
Anti-inflammatory effect of fluoxetine in an OGD/R model of BV-2 cells (**A**) Cell viability was assessed by CCK-8 assay. The x-coordinate represented time points, and the y-coordinate represented the absorbance at 450 nm for the analysis of cell proliferation. (**B**) The levels of TNF-α, IL-1β, and IL-6 in supernatant were examined using ELISA assay. (**C**) The levels of p65 and p50 phosphorylation were detected by western blotting. Total p65 and p50 were detected as reference for phosphorylated p65 and p50. Quantitative analysis and representative images were provided. Tests were carried out on three biological triplicates. *P<0.05; **P<0.01.

Cell viability testing was first conducted for fluoxetine by CCK-8 assay to detect the toxic effect. We found that 20 μM fluoxetine significantly inhibited cell viability compared with the control group, while no significant difference was observed between the 1, 2.5, 5, and 10 μM fluoxetine and the control group (Figure 1A). BV-2 cells were treated with fluoxetine and suffered from OGD/R for 48 h. The levels of TNF-α, IL-1β, and IL-6 in supernatant obtained from the OGD/R group were increased compared with the control group, while the administration of fluoxetine significantly decreased the levels of TNF-α, IL-1β, and IL-6 in supernatant compared with the OGD/R group in a dose-dependent manner (Figure 1B). We then detected whether NF-κB was involved in this process through the analysis of the phosphorylation levels of the two subunits of NF-κB, including p65 and p50. We found that the levels of p65 and p50 phosphorylation were significantly increased compared with the control group, while the administration of fluoxetine largely attenuated those effects in a dose-dependent manner (Figure 1C).

### Administration of fluoxetine enhances IκB-α under OGD/R challenge in protein, but not in mRNA level

**Figure 2:**
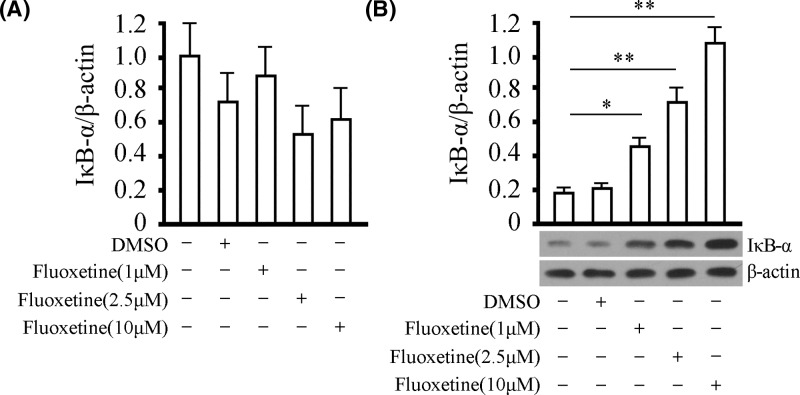
Effect of fluoxetine on the level of IκB-α under OGD/R (**A**) The level of IκB-α in mRNA was detected by real-time PCR in BV-2 cells under OGD/R. Quantitative analysis was provided for the level of IκB-α in mRNA in different groups. (**B**) The level of IκB-α in protein was detected by western blotting in BV-2 cells under OGD/R. Quantitative analysis and representative images were provided for the level of IκB-α in protein in different groups. Tests were carried out on three biological triplicates. *P<0.05 versus DMSO group, **P<0.01 versus DMSO group.

It is acknowledged that IκB-α usually binds to two subunits of NF-κB to form a trimer, reducing the phosphorylation of p65 and p50, thus keeping NF-κB inactivated. We then detected the effect of fluoxetine on the level of IκB-α. Fluoxetine was given at different doses for 48 h under OGD/R and the levels of IκB-α in mRNA and protein were examined. No significant difference in IκB-α in mRNA level was observed amongst the groups with or without the treatment of fluoxetine. However, the administration of fluoxetine significantly increased the level of IκB-α in protein in a dose-dependent manner (Figure 2A,B). These results indicated that the anti-inflammatory effect of fluoxetine was participated by IκB-α through the influence of the level of IκB-α in protein instead of mRNA.

### Fluoxetine inhibits the ubiquitylation of IκB-α in BV-2 cells

**Figure 3:**
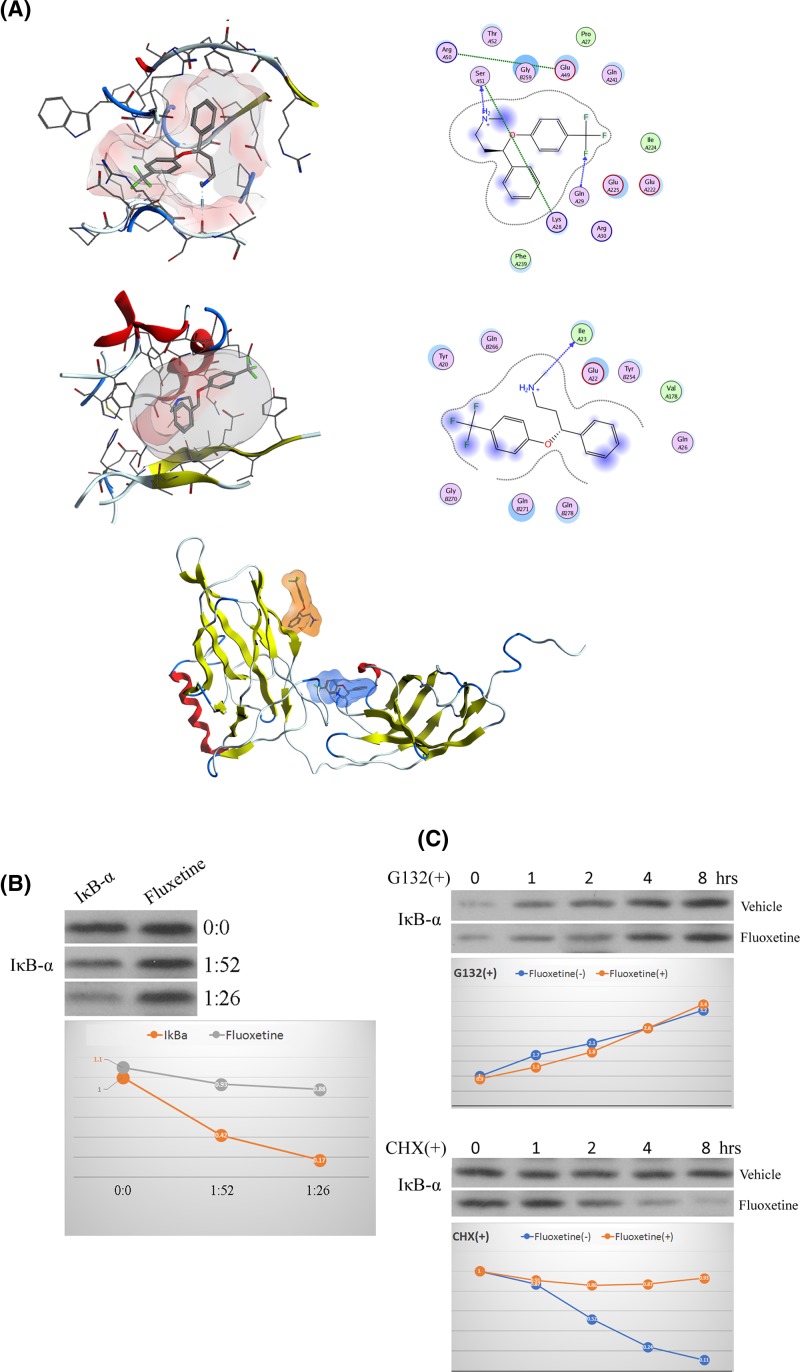
Effect of fluoxetine on the ubiquitylation of IκB-α in BV-2 cells (**A**) The structure of full-length IκB-α complex with fluoxetine. Top: potential binding site 1, with a binding energy of −7.3 kcal/mol, including hydrophobic and hydrogen bond interaction. Fluoxetine provided a hydrogen bond acceptor and donor with a strong hydrogen bond to ser51; middle: potential binding site 2, with a binding energy of −6.6 kcal/mol, included hydrophobic and hydrogen bond interaction. Fluoxetine provided a hydrogen bond donor; bottom: overall view of the combination of IκB-α and fluoxetine; blue, potential binding site 1; orange, potential binding site 2. (**B**) Detection of protein content after enzymatic hydrolysis. Left: optical density change of targetted bands in different the concentrations of pronase; right: scanned image of target bands. (**C**) Protein half-life detection. Left: change of IκB-α at different times with the administration of G132; right: change of IκB-α at different times with the administration of CHX.

According to the results obtained in Figure 2, the administration of fluoxetine increased the level of IκB-α in protein but not in mRNA, indicating that fluoxetine might not suppress the production of IκB-α. We then detected whether fluoxetine could effect the ubiquitylation of IκB-α in BV-2 cells, an enzymatic post-translational modification, through the approach of molecular docking, which was widely used in drug structure design and target prediction with high accuracy. We found two potential fluoxetine binding sites on IκB-α, with a binding energy of −7.3 kcal/mol and −6.6 kcal/mol, respectively (Figure 3A). The former binding site was involved in hydrophobic and hydrogen bond interaction, with fluoxetine providing a hydrogen bond acceptor and a hydrogen bond donor, forming a strong hydrogen bond with ser51. The latter binding site also included hydrophobic and hydrogen bond interaction for the combination of fluoxetine. To verify the direct binding of fluoxetine to IκB-α, we used a modified DARTS assay to investigate whether fluoxetine and IκB-α bond to each other directly. As shown in Figure 3B, the undigested proteins increased pronase in the system in the vehicle group, while no significant changes were observed in the level of the undigested proteins with the administration of fluoxetine. In addition, G132 (50 μM) and cycloheximide (CHX; 100 μg/ml), inhibitors of protein biosynthesis in cells, were used to inhibit the production of proteins. The level of IκB-α was similarly increased in a time-dependent manner under the stimulation of G132 with or without fluoxetine. However, under the challenge of CHX, the level of IκB-α was decreased in a time-dependent manner with the administration of fluoxetine, but not in the group without fluoxetine (Figure 3C). These results suggested that fluoxetine directly binded to IκB-α and thus inhibited its ubiquitylation.

### Genetic intervention through a lentiviral approach

**Figure 4:**
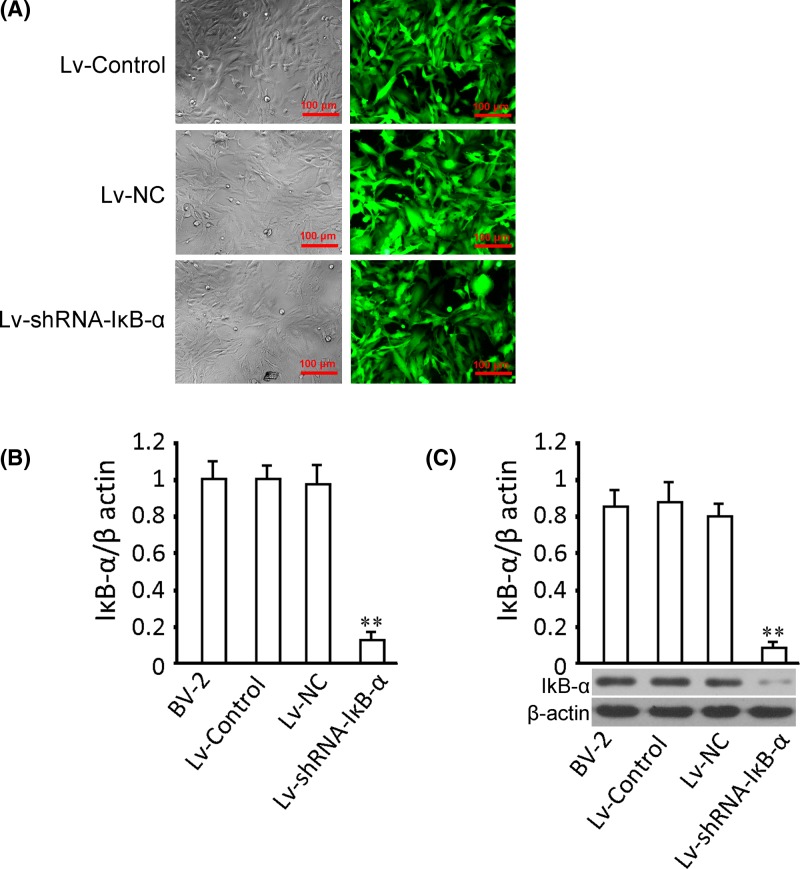
Infection of BV-2 cells and quantification of IκB-α level in mRNA and protein (**A**) After transfection for 72 h, GFP expression of Lv-Control, Lv-NC, and Lv-shRNA-IκB-α transfecting groups showed a gene delivery efficiency close to 100%. Top: dark field, bottom: corresponding fluorescence field. Gene delivery efficiency was estimated by accounting the ratio of cells expressing GFP of the total cell number. MOI = 20; magnification, 120×. (**B**) Quantitative analysis of the level of IκB-α in mRNA with or without transfection. (**C**) Representative images and quantitative analysis of the level of IκB-α in protein with or without transfection. Tests were carried out on three biological replicates, *P<0.05 versus Lv-NC; **P<0.01 versus Lv-NC.

To knock down target genes in validating the specificity of the pathway, we constructed the transfection of the recombinant virus Lv-shRNA-IκB-α and its control virus (Lv-NC and Lv-Control). Green fluorescence was detected for the determination of successful transfection of Lv-Control, Lv-NC, and Lv-shRNA-IκB-α. We found that after transfection for 72 h, green fluorescence was observed in most BV-2 cells, with the gene delivery efficiency close to 90% in all three groups (Figure 4A,B). Compared with the Lv-NC group, cells transfected with Lv-shRNA-IκB-α showed a significant decrease in the level of IκB-α in mRNA. Similar trends of changes in the level of IκB-α in protein was shown (Figure 4C). These results indicated succeeding in intervening the expression of IκB-α.

### Effect of IκB-α knockdown on fluoxetine-mediated suppression of inflammatory reaction under the challenge of OGD/R

**Figure 5:**
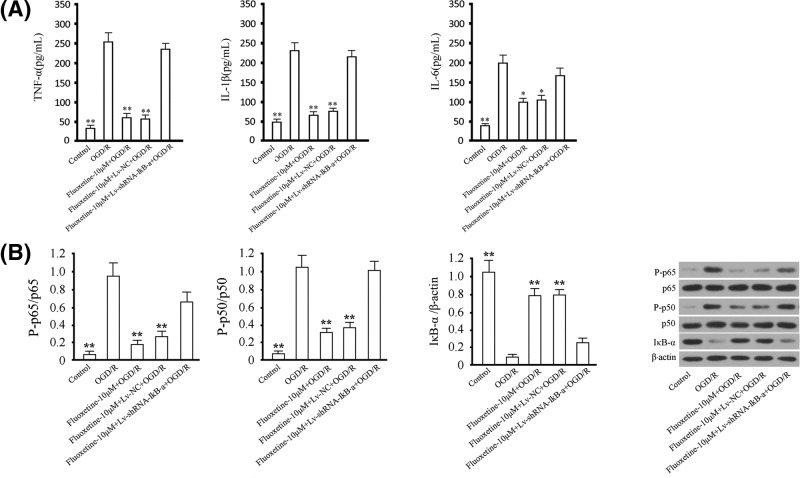
Involvement of IκB-α in fluoxetine-mediated suppression of inflammatory reaction under the challenge of OGD/R (**A**) The levels of TNF-α, IL-1β, and IL-6 in supernatant in BV-2 cells were examined using ELISA assay. (**B**) Phosphorylated of p65 and p50 and IκB-α were detected by western blotting. Total p65 and p50 were detected as reference for phosphorylated p65 and p50, and β-actin for IκB-α. Quantitative analysis and representative images were provided. Tests were carried out on three biological triplicates.*P<0.05 versus the control group; **P<0.01 versus OGD/R group.

According to the data provided above, fluoxetine suppressed inflammatory reaction in BV-2 cells under the challenge of OGD/R through the inhibition of IκB-α-mediated NF-κB activation. We finally detected the influence of knocking down IκB-α on the fluoxetine-mediated anti-inflammatory effect. We found that the levels of TNF-α, IL-1β, and IL-6 in supernatant from BV-2 cells were increased under the challenge of OGD/R, while the administration of fluoxetine alleviated the increasing effect (Figure 5A). The suppressive effect was significantly attenuated by the transfection of Lv-shRNA-IκB-α. In addition, the levels of p65 and p50 phosphorylation were significantly increased in BV-2 cells under the challenge of OGD/R and the level of IκB-α was significantly decreased. The administration of fluoxetine increased the level of IκB-α and suppressed the phosphorylation levels of p65 and p50 compared with the OGD/R group. However, those effects of fluoxetine were largely attenuated by the transfection of Lv-shRNA-IκB-α (Figure 5B).

## Discussion

It is acknowledged that MG extensively distribute in the central nervous system, accounting for 20% of the total number of brain gliocytes [29]. In normal condition, MG remains at resting status with multiple forms. They are greatly activated on the occurrence of cerebral ischemia, for the triggering of inflammatory responses [30]. Through multiple-session monitoring of inflammation cytokines in cerebrospinal fluid and blood of patients with ischemic stroke, researchers found that MG could be activated within hours and recruited to the infarct lesion [31]. Those findings provide direct evidence for MG activation in ischemia/reperfusion, which serves as an important target for the development of drugs against ischemic stroke [32,33]. For the study of potential mechanisms in the process MG activation, a previous study demonstrated that the MAPK-mediated signaling pathway was involved in this process through which the NF-κB-related signaling was triggered, leading to the secretion of several proinflammatory cytokines including TNF-α, IL-1β, and IL-6 [34].

Development of anti-inflammatory drugs targetted on MG activation has become a hotspot in research on cerebral ischemic injury. Acute inflammation has been demonstrated to play a critical role in the secondary brain injury induced by cerebral ischemia/reperfusion, and the inflammatory reaction induced by inflammatory cells contributed to the delayed neuronal death [35–37]. As a result, anti-inflammatory drugs could be used to treat delayed neuronal damage caused by cerebral ischemia [38–40]. However, so far, such agents are seldom available in the treatment of ischemic stroke. The anti-inflammatory effect of fluoxetine related to suppression of NF-κB activity was conducted in an LPS-induced inflammatory model [41]. Here, we detected the function of fluoxetine in an OGD/R model. In the present study, we first ran a cell viability test in BV-2 cells under the challenge of OGD/R so as to choose the doses of fluoxetine in use in the safe range. We then showed an anti-inflammatory effect of fluoxetine in BV-2 cells under the challenge of OGD/R through the inhibition of the levels of TNF-α, IL-1β, and IL-6 in a dose-dependent manner at the range of 1–10 μM. In addition, the administration of fluoxetine significantly attenuated the increase in p65 and p50 phosphorylation compared with the OGD/R group, indicating that the anti-inflammatory effect of fluoxetine might be associated with the regulation of NF-κB activity. Those findings were in accordance with previously related studies on fluoxetine, showing the anti-inflammatory effect of fluoxetine in ischemic stroke [42,43].

We then investigated the specific mechanisms. Since enhancing the level of IκB plays a suppressive effect on inflammatory reaction induced by the formation of IKKB/IKK-KKB/IκB complex, here we detected the interaction between fluoxetine and IκB-α. Although fluoxetine was previously reported to produce an inhibiting effect on the activation of MG in ischemic stroke through suppression of NF-κB-related signaling, no direct evidence as well as molecular mechanism has been provided [24]. Here in our current study, we for the first time provided the direct evidence on the combination of fluoxetine and IκB-α. In our study, two potential fluoxetine binding sites on IκB-α were found through DARTS experiments and the interaction between fluoxetine and IκB-α led to the inhibition of IκB-α ubiquitylation, thus leading to the increase in IκB-α and the subsequent suppression of NF-κB activity. We then transfected Lv-shRNA-IκB-α as well as its negative control lentivirus for the knockdown of IκB-α in BV-2 cells under the challenge of OGD/R. We found that knocking down the expression of IκB-α largely attenuated the suppressive effect of fluoxetine as well as effect on NF-κB-related signaling in BV-2 cells under the challenge of OGD/R. Furthermore, since no significance was found between the OGD/R group and OGD/R group with the treatment of fluoxetine and Lv-shRNA-IκB-α transfection, we thought that the anti-inflammatory effects of fluoxetine was to a large extent mediated by the regulation of IκB-α signaling.

## Conclusion

Taken together, in the present study, we uncovered the anti-inflammatory effect of fluoxetine in MG under the challenge of OGD/R, an *in vitro* model of cerebral ischemia/reperfusion, as well as its specific mechanism related to the regulation of NF-κB-mediated signaling. We believe that our findings might provide strong evidence on the application of fluoxetine in the treatment of ischemic stroke taking advantage of its anti-inflammatory effect.

## Abbreviations

BCA: bicinchoninic acid
CCK-8: cell counting kit-8
CHK: cycloheximide
DARTS: drug affinity responsive target stability
DMEM: Dulbecco’s modification of Eagle’s medium
ELISA: enzyme-linked immunosorbent assay
FCS: fetal calf serum
GFP: green fluorescent protein
MG: microglia
NC: negative control
OGD/R: oxygen glucose deprivation/reoxygenation
